# Supplementary material for: Evidence of Long-Lasting Humoral and Cellular Immunity against SARS-CoV-2 Even in Elderly COVID-19 Convalescents Showing a Mild to Moderate Disease Progression
Source: Life (Basel). 2021 Aug 9;11(8):805. doi: 10.3390/life11080805 (PMC8401673; doi:10.3390/life11080805)
Supplement: Supplementary file 1 [file life-11-00805-s001.zip › life-1314911-supplementary.pdf]

Supplementary Materials:

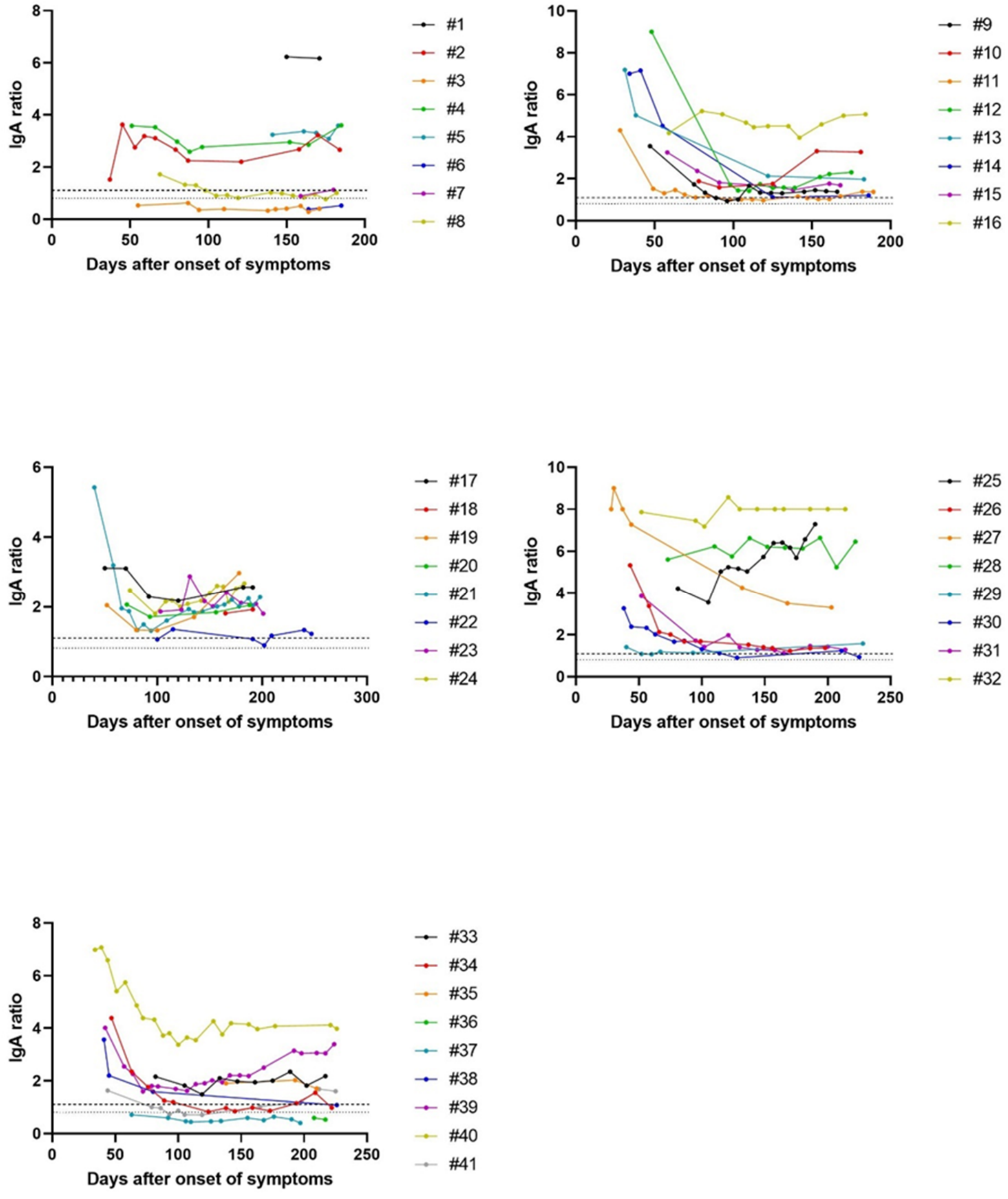

Figure S1. Persistence and decay of anti-SARS-CoV-2 IgA antibodies in COVID-19 convalescents (n = 41). Anti-SARS-CoV-2 IgA antibody expression of convalescents at the indicated days after onset of symptoms. Semi-quantitative ratios were determined using the ELISA-based Euroimmun assay. According to the manufacturer, values above the upper dashed line (ratio = 1.1) are considered as seropositive, and values below the lower dashed line (ratio = 0.8) are considered as seronegative. Values between the horizontal dashed lines were interpreted as equivocal–positive.

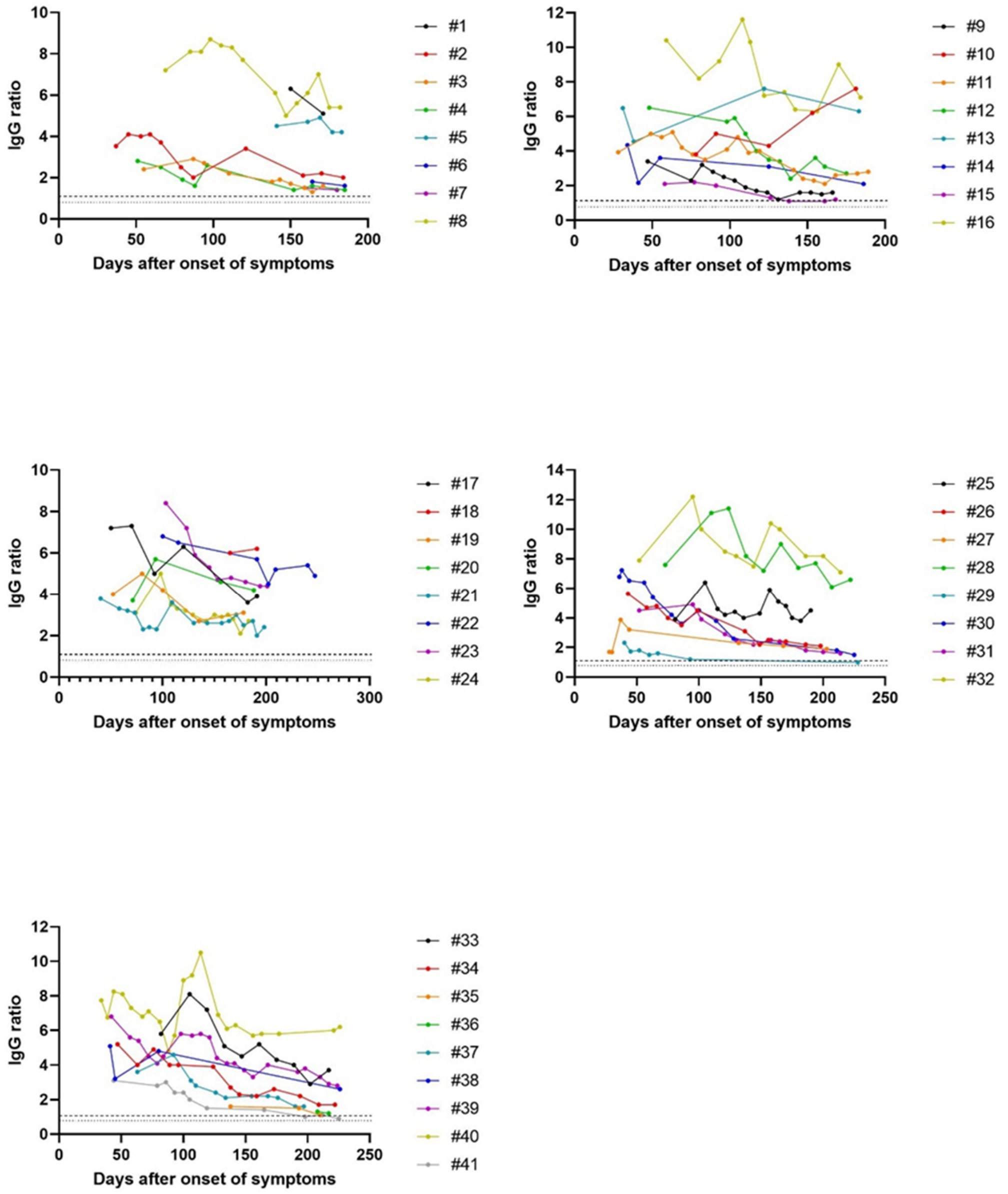

Figure S2. Persistence and decay of anti-SARS-CoV-2 IgG antibodies in COVID-19 convalescents (n = 41). Anti-SARS-CoV-2 IgG antibody expression of convalescents at the indicated days after onset of symptoms. Semi-quantitative ratios were determined using the ELISA-based Euroimmun assay. According to the manufacturer, values above the upper dashed line (ratio = 1.1) are considered as seropositive, and values below the lower dashed line (ratio = 0.8) are considered as seronegative. Values between the horizontal dashed lines were interpreted as equivocal–positive.

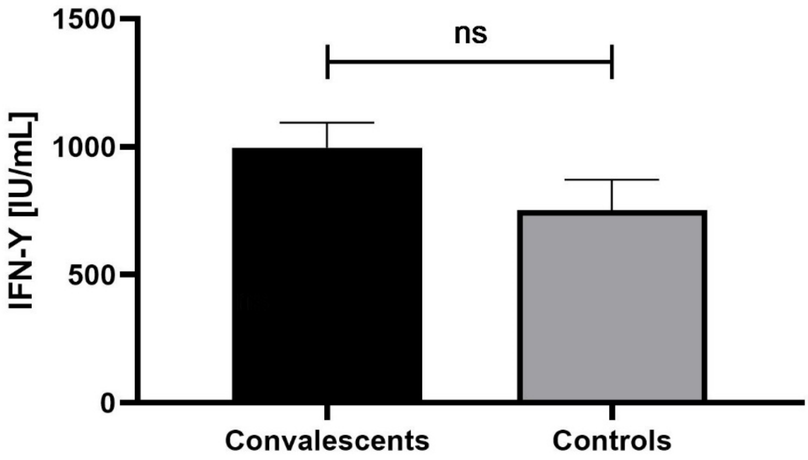

Figure S3. IFN-γ release after stimulation of whole blood of convalescents (n = 41) and controls (n = 18) with LyoSpheres pellets. Cells in our study were stimulated in a parallel setup using LyoSpheres pellets containing a CD3 T-cell receptor agonist and a viral TLR 7/8 ligand (resiquimod or R848) for assay-validation and as a positive control. A comparatively high IFN-γ release was detected because the QuantiFeron-assay was originally designed and optimized for the use of LyoSpheres. Nevertheless, no significant differences in the IFN-γ secretion was detected after stimulation of the whole blood of COVID-19 convalescents and healthy controls. ns: not significant.
